# Cerebral phaeohyphomycosis due to *Cladophialophora bantiana* in an immunocompetent individual: A case report and brief review of literature 

**DOI:** 10.18502/CMM.6.2.2693

**Published:** 2020-06

**Authors:** Prachala G Rathod, Bibhabati Mishra, Archana Thakur, Poonam S Loomba, Abha Sharma, Ashish Bajaj, Madhusmita Das, Ashna Bhasin

**Affiliations:** 1 Department of Microbiology, Govind Ballabh Pant Institute of Postgraduate Medical Education and Research, New Delhi, India

**Keywords:** Brain abscess, Phaeoid fungi, Voriconazole

## Abstract

**Background and Purpose:**

Fungal infections of the central nervous system (CNS) are life-threatening conditions that are frequently misdiagnosed with bacterial and viral CNS infections. Cerebral phaeohyphomycosis is a cerebral infection caused by dematiaceous fungi, especially *Cladophialophora bantiana*. Very few cases of fungal CNS infection have been reported across the world. High clinical suspicion should be cast for the patients with brain abscess that do not respond to conventional antibiotic therapy.

**Case report:**

We report a case of a 21-year-old male presenting with headache, seizures and weakness in the limbs. Radiological examination revealed multiple brain abscesses. After surgical excision and laboratory evaluation, it was found to be caused by C. bantiana. The patient’s outcome was good with surgical excision and voriconazole therapy.

**Conclusion:**

Brain abscess caused by *C. bantiana* is on rise, especially in immunocompromised groups. Thus, high clinical suspicion, accurate diagnosis and management are the fundamentals for good prognosis.

## Introduction

Fungal infections of the central nervous system (CNS) are rare clinical entities that are associated with significant morbidity and mortality. Clinically, CNS fungal infections present most commonly as intraparenchymal abscess (87%) and meningitis (10%) [ 1 ]. Cerebral phaeohyphomycosis, also referred to as black molds and phaeoid fungi, is a cerebral infection caused by dematiaceous or melanized fungi and their relatives [ 2 ]. The black yeast-like fungi causing cerebral phaeohyphomycosis are *Cladophialophora bantiana, Rhinocladiella mackenziei, Verruconis gallopavum, Bipolaris spicifera, Fonsecaea pedrosoi, Chaetomium strumarium, Exophiala dermatitidis,* and *Acrophialophora fusispora*. Among this group, *C. bantiana* is the most common (48%) cause of cerebral phaeohyphomycosis [ 1 , 3 - 9 ]

*Cladophialophora bantiana* is ubiquitously a soil pathogen that is found worldwide, most prevalently in Asian countries like India. It is a highly neurotropic fungus. The clinical presentation of CNS infection mimics space-occupying lesions like malignancy or tuberculoma [ 10 ]. These nonspecific symptoms make the diagnosis difficult, especially in immunocompetent hosts where the lack of predisposing factors obscures the diagnosis. In such a scenario, laboratory-based investigations play a vital role in the diagnosis and management of such dreadful infections.

## Case report

A 21-year-old male metal company worker referred to the neurosurgery department with the complaints of chronic headache and generalized tonic-clonic seizures for 3 years, as well as weakness in the left upper and lower limbs, diplopia, and projectile vomiting for 1 month. There was no history of chronic fever, trauma, surgery, and any other major illnesses (e.g., diabetes or hypertension). The patient was prescribed with anti-tuberculosis drugs by a private medical practitioner a year earlier although no reports were suggestive of diagnosing tuberculosis.

On physical examination, the patient was afebrile, conscious, and well oriented. His vitals were normal with a pulse rate of 90/min, blood pressure of 110/80 mmHg, and respiratory rate of 18/min. The power was reduced in the left upper and lower limbs (grade 4/5). The pupils of both sides were of normal size and normally reacted to light. Other parameters in the physical examination were unremarkable. All hematological investigations were within the normal limits, except for white blood cell count (16,400 cells/µl) and polymorphonuclear leukocytosis (showing toxic granulations).

**Figure 1:**
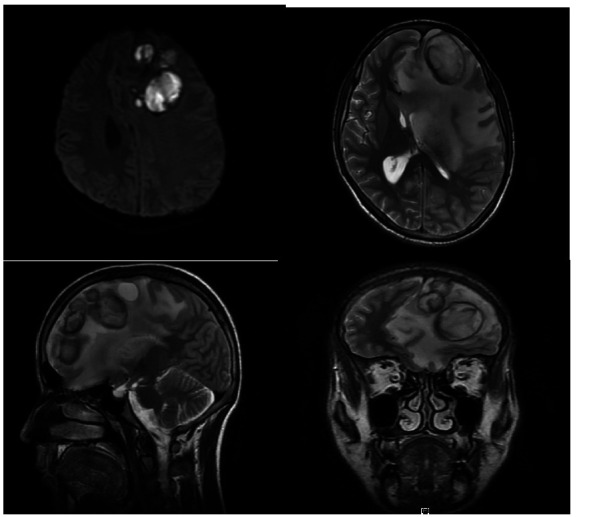
Magnetic resonance imaging in different planes showing multiple cerebral abscesses

With regard to the complaints and physical examination, the patient was subjected to contrast-enhanced computer tomography scan (CECT) and magnetic resonance imaging (MRI). The CECT showed multiple ring-enhancing lesions in the left frontal lobe. The MRI demonstrated multiple heterogeneous focal lesions in the frontal cortex with marked perilesional edema causing mass effect and compression of the left lateral ventricle with a rightward midline shift (Figure 1). Imaging modalities and clinical presentation were both suggestive of left frontal cerebral abscess.

Therefore, the patient underwent left frontal craniotomy. Intraoperatively, three well-encapsulated abscesses were observed in the left frontal cerebral lobe just anterior to the coronal suture. Around 60 to 70-ml thick green colored exudate was aspirated from the abscess cavity, followed by complete excision and evacuation of the abscesses. The aspirated exudate was sent to the laboratory for examination. The patient was started on ceftriaxone (2 gm) and metronidazole (100 µl) intravenous injection, as well as an anticonvulsant (i.e., levetiracetam), till the culture reports were available.

**Figure 2:**
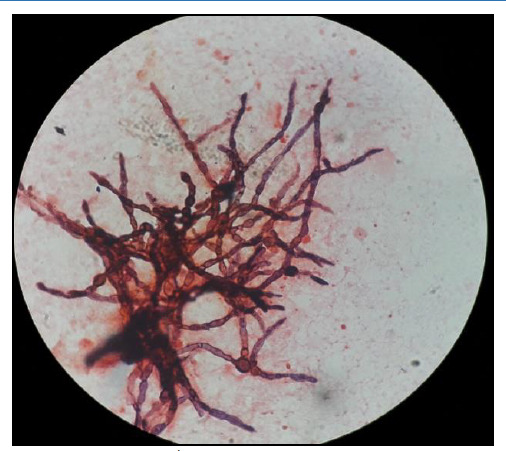
Gram’s Stain

**Figure 3:**
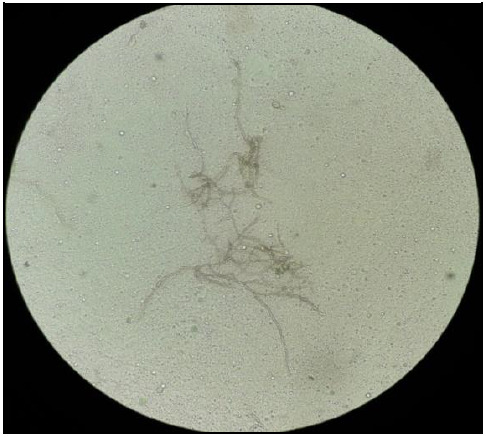
Potassium hydroxide mount

The brain abscess exudate was received in the Department of Microbiology for microscopy and culture. On Gram stain, Ziehl Neelsen stain (20%, 1% H2SO4), and KOH mount, few pus cells and septate hyphae bearing chains of conidia were seen with no evidence of any bacteria (Figures 2 and 3). The laboratory personnel alerted the surgeons about the findings with the advice of starting antifungal therapy.

The specimen was inoculated onto culture media for both bacterial and fungal isolation. For bacterial culture, blood agar and Mackonkey’s agar (HiMedia, Mumbai, India) were inoculated and incubated at 37°C for 48 h. Sabouraud’s dextrose agar (SDA) plain and SDA (HiMedia, Mumbai, India) with gentamicin and actidione were incubated for 14 days at 37°C and 25°C for fungal culture.

**Figure 4:**
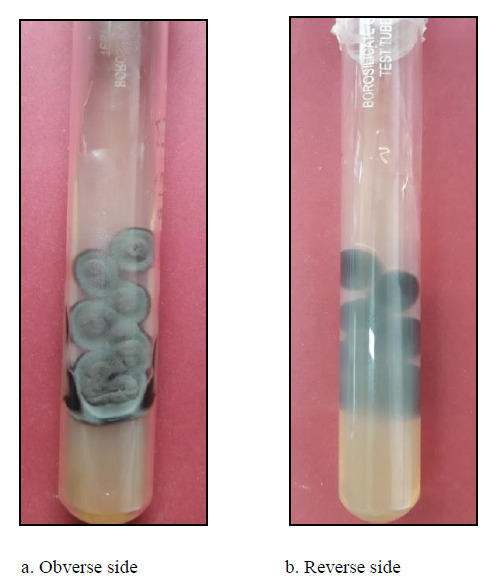
Culture on SDA (a,b) and Blood agar (c)

The blood agar and Mackonkey’s agar were sterile for bacterial growth after 48 h of inoculation. However, the media were further incubated at 37°C for a week. On days 5 and 10, a 5-mm, olivaceous grey, suede, or velvety fungal colony was observed on blood agar and SDA, respectively (Figure 4). A lactophenol cotton blue mount showed pale brown, septate hyphae with many broken chains of single-celled, oval conidia. Presumptive identification of* Cladophialophora *was made based on this macroscopic and microscopic morphology. In addition, slide culture and few physiological tests were employed to confirm the isolate. For urea hydrolysis, Christensen’s urea agar (HiMedia, Mumbai, India) was incubated at 30°C for 10 days. Similarly, thermotolerance was assessed by incubating the fungal isolate at 40-42°C for 3 weeks. 

**Figure 5:**
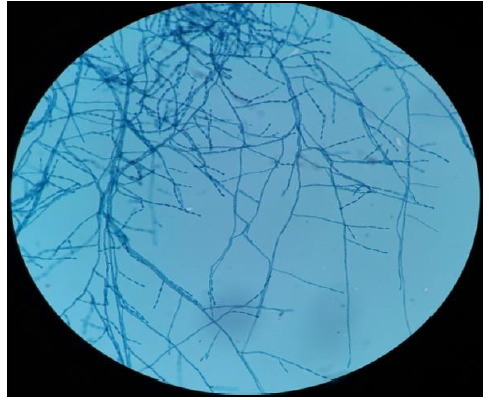
Lactophenol Cotton Blue Microscopy

The slide culture lactophenol cotton blue mount revealed hyphae with poorly differentiated conidiophores-bearing long, sparsely branched chains of single-celled, oval conidia with acropetal arrangement (Figure 5). The urea hydrolysis was positive, and the strain was thermotolerant and cycloheximide resistant. On the basis of the above features, the strain was identified as *C. bantiana*. The strain was also confirmed based on the morphological characteristics in the Department of Microbiology, All India Institute of Medical Sciences, New Delhi. 

The focus of fungal infection in this patient could not be determined as blood, urine, tracheal secretions, and CSF cultures were negative. In addition, there was no evidence of any type of immunosuppression in the patient. Based on the laboratory reports, the patient was started on oral voriconazole (200 mg). His headache and seizures showed improvement; therefore, he was discharged with the continuation of antifungal and anticonvulsant agents. The patient has been on regular follow-up to date. His recovery has been uneventful, except for a few episodes of seizures. After 6 months, his MRI did not show any evidence of brain lesions.

**Ethical considerations**

The current study was approved by the Ethics Committee of Govind Ballabh Pant Institute of Postgraduate Medical Education and Research (Ethics Committee code: 2019/09/05-03)

## Discussion

Brain abscess with a fungal etiology is a very rare condition, accounting for just 2% of the brain abscesses [ 11 ]. Phaeohyphomycosis represents an infection with melanized fungus and their relatives.

Melanin, a pigment formed during the oxidative polymerization of phenolic compounds, allows the fungus to evade the host’s immune response, thereby protecting the fungal cell from antifungal agents [ 12 ]. In phaeohyphomycetes, *C. bantiana* is the most common fungus causing brain abscess. 

The high affinity of this fungus towards the glial tissue is accountable for its exclusive neurotropism [ 13 ]. Besides melanin production as a potential virulence marker, thermotolerance at higher temperatures (&gt;40°C) is also considered to be its pathogenicity and survival marker [ 14 ]. The hot humid climate of tropical regions, like India, sets the ground for this fungus to thrive.

Since *C. bantiana* is a ubiquitous soil and plant pathogen, its modes of transmission include the inhalation of conidia, direct extension from paranasal sinuses, and accidental trauma to the brain [ 11 ]. Though the nature of occupation affects the infection mode of transmission, it could not be ruled out in our case. Since our patient was a metal factory worker, we assumed that he must have either received some inconspicuous trivial injuries or inhaled fungal spores causing the haematogenous spread of the fungus, leading to cerebral abscess.

Brain abscess due to *C. bantiana* occurs in both immunocompetent and immunocompromised groups though the prevalence is slightly higher in the former group due to unknown reasons [ 15 ]. Our case was also an immunocompetent host. A few case reports and case series from India have also reported similar findings [ 1 , 16 ]. The clinical presentation of fungal brain abscess is nonspecific; however, its symptoms mimic those of other space-occupying lesions like tumors and tuberculoma, as well as those caused by microbial infections [ 13 ]. 

The most common symptoms of this condition include headache, fever, hemiparesis, altered sensorium, aphasia, visual disturbances, and vomiting [ 15 ]. Our case also presented with insidious headache, hemiparesis, and vomiting as major complaints. A few case studies have also reported similar symptoms [ 1 , 10 , 17 ]. However, most of the time, the uncertainty of this clinical scenario causes a delay in diagnosis, especially in immunocompetent individuals in whom fungi as the etiology of brain lesions is rarely considered.

The number of cases with* Cladophialophora *species has risen exponentially in the past few decades, especially in Asian countries like India [ 15 ]. This species has been also declared as an emerging pathogen in animals [ 18 ]. The cerebral phaeohy-phomycosis infections have a poor prognosis and are mostly associated with fatal outcomes. Therefore, it is required to cast a timely suspicion on the fungal etiology of brain abscess and implement appropriate laboratory investigations with special consideration of fungal culture.

Recent re-evaluation of* Cladophialophora *genus has been performed by multilocus sequencing giving seven different species. Within* Cladophialophora *genus, in addition to *C. bantiana*, *C. modesta* is also a neurotropic fungus [ 12 ]. However, its inability to grow at higher temperatures can differentiate it from the former species. In our case, the identification of the causative fungus was based on its typical morphology, attributes of thermotolerance and source of the clinical specimen. 

Though culture is the gold standard method for the diagnosis of fungal CNS infection, it is time consuming and laborious and has safety issues of laboratory personnel. Many new diagnostic methods, such as polymerase chain reaction [ 19 ], rolling circle amplification [ 11 ], matrix-assisted laser desorption/ ionization-time of flight [ 20 ], and amplified fragment length polymorphism analysis, have evolved in recent years [ 21 ]. In our case, the diagnosis was based on culture only due to the unavailability of other diagnostic methods. A few case reports have also identified *C. bantiana* based on the culture morphology [ 22 , 23 ].

There are not any established traditional guidelines for the management of this infection by introducing the proper type of surgery, antifungal agent, and treatment duration. When surgical management is concerned, a complete resection of brain abscess is considered to be associated with better outcomes than partial excision and simple aspiration [ 11 ]. The recommended medical therapy is either monotherapy or combination of antifungal therapy. Earlier amphotericin B was considered the drug of choice. However, few cases have reported resistance and treatment failure with amphotericin monotherapy [ 1 ]. Voriconazole demonstrates a good in-vitro activity against *C. bantiana* and has good cerebrospinal fluid penetration. This agent is used in many cases of cerebral phaeohyphomycosis with a survival rate of 50% for 2 years. Pasoconazole and itraconazole also exhibit good in-vitro activity against *C. bantiana* [ 14 ].

In the present case, voriconazole was administered for 6 months. Due to the unavailability of the facility required for antifungal susceptibility testing (containment level 3), the choice of antifungals was based on previous successfully treated cases published in the literature. Our case survived with complete surgical resection and voriconazole therapy for 6 months. Many studies report similar successful outcomes [ 19 , 24 , 25 ]. In the present study, the patient was postoperatively followed up for a one-year period and showed no casualty, except for a few episodes of seizures. 

Chakrabarti et al. [ 15 ] performed a systematic review on 124 cases of brain abscess caused by *C. bantiana* in India and other countries taking into account the clinical characteristics, management, and outcome. Similarly, Suri et al. [ 23 ] conducted a review on 28 cases of *C. bantiana* brain abscess in India from 1962 to 2009. Table 1 presents a few studies on brain abscess caused by *C. bantiana* carried out in India and other countries highlighting the major clinical features, associated risk factors, diagnostic method, and therapeutic techniques.

**Table 1:** Case reports of brain abscess due to *Cladophialophora bantiana*

Reference number	Age/gender	Clinical features	Risk factor	Diagnostic modality	Therapy	Outcome
Indian scenario						
Suri P. et al., [23], 2010	65/M	Left hemiparesis, slurring of speech, altered sensorium	None	Culture	Total excision, Amphotericin B, Voriconazole	Expired
Aher A. and Rastogi V. [25], 2012	55/M	Slurred speech, weakness	Diabetes mellitus	Culture	Craniotomy, Amp-B	Expired
Agrawal A. et al., [10], 2014	45/F	Headache, weakness, vomiting, slurred speech	None	Histopathology	Craniotomy, antifungal	Expired
Kumar D. et al., [26], 2016	57/M	Headache, vomiting, behavioral changes	Diabetes mellitus	Histopathology	Excision, Fluconazole	Survived
Jangla S. M. and Vishwanathan I [27], (2017)	55/M	Slurred speech, diplopia, memory loss	Diabetes mellitus, hypertension	Culture, Histopathology	Craniotomy, Voriconazole	Survived
Gopalkrishnan R. et al., [24], 2017	69/M	Lower limb weakness	Hypertension	Culture, Histopathology, DNA sequencing	Craniotomy with partial excision, Voriconazole	Survived
Gopalkrishnan R. et al., [24], 2017	65/M	Seizures, altered sensorium	Chronic renal failure	Culture	Stereotactic aspiration, Voriconazole	Survived
World Scenario						
Revankar S. G., [1], 2011	79/F	Weakness	Hypertension deep venous thrombosis, pulmonary embolus	Culture	Excision, Flucytosine, Voriconazole, Amp-B	Expired
Huang Wen M. et al., [28], 2011	38/M	Seizures, hemiparesis	NIDDM, myelodysplasia	Culture, DNA sequencing	Burr hole aspiration, Amp-B	Expired
Na-Young Jung et al., [8], 2014	75/M	Poor cognition, memory loss	None	Culture, histopathology	Excision, Voriconazole	Survived
Liutkus D. et al., [29], 2016	62/F	Headache, unconsciousness, hemiparesis	None	Histopathology	Excision, Fluconazole	Survived
Kuan C. S. et al., [30],2016	49/M	Seizures, fever, headache, weakness	None	Culture, multilocus phylogenetic analysis	Excision, Amp-B, Itraconazole	Survived
Khaliq M. F. et al., [11], 2019	64/M	Confusion, staggering gait	Chronic smoker, pulmonary nocardiosis	Culture	Craniotomy with partial excision, Amp-B, Voriconazole	Unknown
Present study	21/M	Headache, seizures, diplopia, weakness, vomiting	None	Culture	Excision, Voriconazole	Survived

## Conclusion

The occurrence of brain abscess caused by* Cladophialophora *is increasingly reported worldwide both in immunocompetent and immunocompromised hosts. Based on the findings of the current study, it can be concluded that the timely diagnosis and aggressive surgical and medical management of this condition can alter the prognosis of this disease.
